# Metastatic Extramammary Paget’s Disease: Pathogenesis and Novel Therapeutic Approach

**DOI:** 10.3389/fonc.2018.00038

**Published:** 2018-02-16

**Authors:** Keitaro Fukuda, Takeru Funakoshi

**Affiliations:** ^1^Department of Dermatology, University of Massachusetts Medical School, Worcester, MA, United States; ^2^Department of Dermatology, Keio University School of Medicine, Tokyo, Japan

**Keywords:** metastatic extramammary Paget’s disease, HER2–PI3K/ERK signaling, lymphangiogenesis, CXCR4–stromal cell-derived factor-1 axis, CD163^+^M2 macrophage, receptor activator of nuclear factor kappa-B ligand–RANK signaling, mismatch-repair deficient, anti-PD-1 antibody

## Abstract

Extramammary Paget’s disease (EMPD) is a rare, slow-growing, cutaneous adenocarcinoma that usually originates in the anogenital area and axillae outside the mammary glands. EMPD mostly progresses slowly and is often diagnosed as carcinoma *in situ*; however, upon becoming invasive, it promptly and frequently metastasizes to regional lymph nodes, leading to subsequent distant metastasis. To date, several chemotherapy regimens have been used to treat metastatic EMPD; however, they present limited effect and patients with distant metastasis exhibit a poor prognosis. Recently, basic and translational investigative research has elucidated factors and molecular mechanisms underlying the promotion of metastasis, which can lead to targeted therapy-based emerging treatment strategies. Here, we aim to discuss current therapies and their limitations; advancements in illustrating mechanisms promoting invasion, migration, and proliferation of EMPD tumor cells; and future therapeutic approaches for metastatic EMPD that may enhance clinical outcomes.

## Invasive Extramammary Paget’s Disease (EMPD) Therapeutic Challenge: Preventing and Treating Tumor Metastasis

Extramammary Paget’s disease is a rare, slow-growing, cutaneous adenocarcinoma that manifests as an erythematous, eczematous plaque outside the mammary gland, occasionally accompanied by hypopigmented patches. EMPD affects apocrine gland-rich sites such as the anogenital area and axillae. Despite requiring broad local resection for the treatment of a primary lesion because of the frequent microscopic extensions and less common satellite lesions beyond the clinical tumor border (1), the prognosis of patients with EMPD is usually good because tumor cells are in the radial growth phase for a prolonged duration, and a majority of cases are treated in the stage of carcinoma *in situ* (2).

However, once EMPD invades into the dermis and becomes invasive EMPD, tumor cells gain high metastatic potential, leading to the development of lymph node (LN) metastasis even in patients with dermal microinvasion (3). Besides, over one-third of patients with LN metastasis consequently develop distant metastasis (4). To date, several chemotherapeutic regimens (Table 1), such as low-dose 5-fluorouracil (5-FU)/cisplatin (FP), FECOM (5-FU, epirubicin, carboplatin, vincristine, and mitomycin C), docetaxel monotherapy, S-1 monotherapy, docetaxel and S-1 combination therapy, and PET (cisplatin, epirubicin, and paclitaxel), have been used to treat metastatic EMPD (5–13); however, few patients overcome tumor recurrence despite tumors in over half of patients’ initially responding to these regimens. In addition, the overall survival (OS) starts declining from 10 months after starting chemotherapy (7), and patients with EMPD with distant metastasis exhibit a poor prognosis with the median OS of 1.5 years and 5-year survival rate of 7% (4). Hence, exploring novel therapeutic strategies to prevent and treat metastatic EMPD is imperative.

**Table 1:** Current systemic therapy for metastatic extramammary Paget’s disease in case studies and case reports.

Reference	No. of patients	Treatment	Type of response	Outcome (months)
Tokuda et al. (5)	22	Low-dose FP (5-FU, cisplatin)	CR (1/22), PR (12/22), SD (6/22), PD (3/22)	PFS: median 5.2, OS: median 12.0
Oashi et al. (6)	7	FECOM (5-FU, epirubicin, carboplatin, vincristine, mitomycin C)	PR (4/7), unevaluable (3/7)	PFS: median 6.5, OS: median 9.4
Yoshino et al. (7)	13	Docetaxel	PR (8/13), SD (3/13), PD (2/13)	PFS: median 7.1, OS: median 16.6
Mikoshiba et al. (8)	1	S-1	PR	PFS: 36
Kato et al. (9)	2	S-1	PR	PFS: 5, 11+
Matsushita et al. (10)	1	Docetaxel + S-1	PR	PFS: 15+
Ogata et al. (11)	1	Docetaxel + S-1	PR	PFS: 12+
Egashira et al. (12)	2	Docetaxel + S-1	CR (1/2), PR (1/2)	PFS: 12, 10, OS: 26, 23
Hirai and Funakoshi (13)	2	PET (cisplatin, epirubicin, paclitaxel)	PR (2/2)	PFS: 14+, 12+
Karam et al. (24)	1	Trastuzumab	PR	PFS: 12
Wakabayashi et al. (27)	1	Trastuzumab	PR	PFS: 12+
Barth et al. (28)	1	Trastuzumab	CR	PFS: 12+
Takahagi et al. (25)	1	Trastuzumab + paclitaxel	PR	PFS: 17, OS: 25
Hanawa et al. (26)	1	Trastuzumab + paclitaxel	PR	PFS: 13
Ichiyama et al. (29)	1	Trastuzumab + paclitaxel	PR	PFS: 24+
Yoneyama et al. (45)	1	Bicalutamide + leuprolide acetate	PR	PFS: 6, OS:14

*CR, complete response; PR, partial response; SD, stable disease; PD, progressive disease; PFS, progression-free survival; OS, overall survival; +, ongoing response; 5-FU, 5-fluorouracil*.

Metastasis is a multistage process that comprises tumor cell invasion, venous/lymphatic intravasation, transit in the vessels, venous/lymphatic extravasation, and proliferation at a new site (14). Recently, some studies have identified that the protein expression of molecules involved in proliferation and survival, including HER2 and mTOR, in the Paget cell (tumor cell of EMPD) is associated with invasiveness, metastasis, and OS (15–17). Similarly, translational research studies have demonstrated that cytokines, chemokines, and immune cells in EMPD confer favorable microenvironment for Paget cells to invade, migrate, and proliferate, thereby promoting metastasis (18–21). The results of current study support that the involvement of both Paget cells and tumor microenvironment factors is essential for metastasis; thus, the understanding of both aspects provides opportunities for the treatment of metastatic EMPD.

## HER2–PI3K/ERK Signaling in EMPD

In the past, several EMPD studies have focused on investigating the HER2–PI3K/ERK signaling in EMPD because it is characterized by the presence of Paget cells, large round, vacuolated, pale-staining cells, as mammary Paget’s disease (MPD) and the clinical success in targeting aberrant receptor tyrosine kinase signaling pathways in breast cancer. Both immunohistochemistry and fluorescence *in situ* hybridization studies of primary and LN metastatic lesions have revealed the HER2 overexpression (HER2 score of 3+ or 2+) in 15–58% of patients with EMPD and that all cases with a HER2 score of 3+ had amplified *ERBB2*, the gene encoding HER2 (16, 22). Of note, a HER2 score of 3+ or 2+ was significantly more common in patients with deeply invasive EMPD that those with *in situ*/superficial invasive (in which tumor invasion was limited to the papillary dermis) EMPD and was correlated with numerous LN metastases (16). Furthermore, about 90% of patients exhibited no difference in the HER2 protein overexpression and *ERBB2* gene amplification between primary tumors and corresponded LN metastasis (23), suggesting that HER2’s contribution to the pathogenesis and progression in a subset of metastatic EMPD and implying the possibility that HER2 blockade disrupts the progression of both primary and metastatic lesions of this population. In corroboration with the hypothesis, six case reports determined that both primary and metastatic lesion of HER2-overexpressed EMPD responded well and attained partial or complete response for 6 months to 2 years by anti-HER2 antibody, trastuzumab alone, or trastuzumab with paclitaxel (Table 1) (24–29). A phase 2 study of trastuzumab with docetaxel for HER2-positive unresectable or metastatic EMPD (UMIN000021311) is ongoing based on these results.

**Figure 1:**
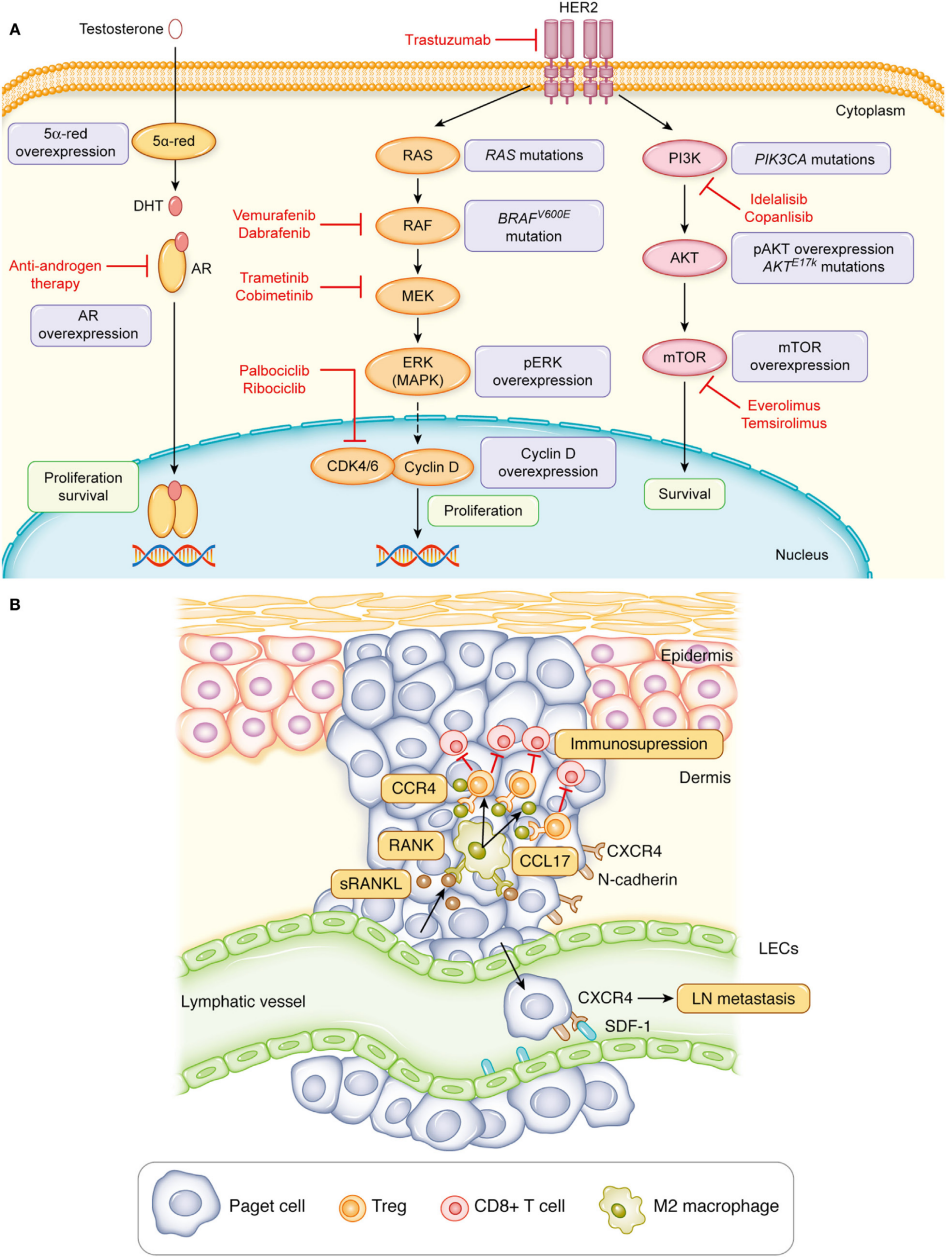
Signaling pathways involved in the progression of extramammary Paget’s disease. **(A)** The aberrant activation of HER2, molecules involved in the RAS–RAF–MEK–ERK signaling or PI3K–AKT–mTOR signaling promote the proliferation and survival of Paget cells. Likewise, the androgen–androgen receptor (AR) signaling can induce the proliferation and survival of Paget cells. Red, Food and Drug Administration-approved drugs for other cancers that target aspects of this pathway. **(B)** The interaction of Paget cells with lymphatic endothelial cells (LECs) through the CXCR4–stromal cell-derived factor-1 (SDF-1) signaling or with CD163^+^Arg1^+^ M2 macrophages through the receptor activator of nuclear factor kappa-B ligand (RANKL)–RANK signaling facilitates metastasis of Paget cells.

HER2 activates several signaling pathways such as the RAS–RAF–MEK–ERK pathway and PI3K–AKT–mTOR pathway, which accelerate cell growth and increase cell survival (30). A study demonstrated that 28 and 56% of HER2-overexpressed EMPD cases presented high expression of phosphorylated ERK and phosphorylated AKT, respectively, with high Ki-67 labeling index (31). However, the high expression of phosphorylated ERK and phosphorylated AKT was also noted in 30 and 33% of EMPD patients without the HER2 overexpression, respectively, but with high Ki-67 labeling index (31). In addition, other studies have demonstrated that Ki-67, as well as mTOR, was expressed at significantly higher levels in invasive cases than carcinoma *in situ* cases (17, 32). Furthermore, the DNA sequencing of EMPD study revealed that 19% of cases comprised mutant *RAS* or *RAF* genes and 35% of cases had mutations in *PIK3CA* (which encodes the catalytic subunit of PI3K) or *AKT1* that activate those pathways (33). In particular, cases with mutations in both *RAS*/*RAF* and *PIK3CA*/*AKT1* signaling pathways were sporadic, and a mutually exclusive pattern was observed (33). Overall, these results highlight that not only HER2 but also the activation of downstream molecules in the RAS–RAF–MEK or PI3K–AKT–mTOR pathways could contribute to the progression of EMPD. Notably, all mutations detected in *RAF* were *BRAF V600E* that can be inhibited by vemurafenib or dabrafenib, the Food and Drug Administration (FDA)-approved drugs for metastatic melanoma (33). In addition, multiple drugs targeting the RAS–RAF–MEK and PI3K–AKT–mTOR pathways have passed through clinical trials and are used for other cancers. Hence, multiple treatment options exist for developing novel therapeutics that target patients with metastatic EMPD with the aberrant RAS–RAF–MEK or PI3K–AKT–mTOR signaling (Figure 1A).

The HER family comprises four type 1 transmembrane tyrosine kinase receptors, HER1 (or EGFR), HER2, HER3, and HER4 (34). Of these, HER2 dimerizes with other members of the HER family, and the dimerization of HER2:HER3 dimers was proven to be the most oncogenic receptor pairing that activates the RAS–RAF–MEK and PI3K–AKT–mTOR pathways in HER2-positive breast cancer cells (35). Hence, pertuzumab, an anti-HER2 antibody that inhibits dimerization with HER1, HER3, and HER4, in combination with trastuzumab and docetaxel attained a significantly longer progression-free survival and OS in untreated HER2-positive metastatic breast cancer compared to trastuzumab plus docetaxel alone; the former regimen is now preferred as the first-line treatment of patients with HER2-positive metastatic breast cancer (36, 37). However, the expression of HER1, HER3, and HER4 has never been detected in EMPD as well as in MPD, and targeting the dimerization of HER2 is unlikely to prove clinical significance in HER2-positive metastatic EMPD (38, 39).

## Hormone Receptors Signaling in EMPD

Signaling through hormone receptors contributes to the tumor development and progression in some cancer types such as breast cancer. More than two-thirds of breast cancers express estrogen receptor (ER), and research has proved that ER-targeted therapy reduces relapse and improves the OS in advanced breast cancer (40). However, MPD expresses ER only in 10% of cases despite being one of the types of breast cancer. Similarly, EMPD also demonstrates a low ER-positive rate at 4% (41). In fact, both MPD and EMPD exhibit a high androgen receptor (AR)-positive rate at 54–90%. In addition, another study reported that the AR expression intensity was significantly higher in invasive EMPD than noninvasive EMPD (41–43). Furthermore, the expression intensity of 5α-reductase and 17β-hydroxysteroid dehydrogenase type 5, enzymes producing androgen, was higher in invasive EMPD than noninvasive EMPD, suggesting the possibility of androgen amplification and the association of the androgen–AR signaling with the progression of EMPD (Figure 1A) (43, 44). In corroboration with the hypothesis, one case report demonstrated that the combined androgen blockade (CAB) therapy by bicalutamide (an anti-androgen drug) and leuprolide acetate (LH–RH agonist), used in the treatment of prostate cancer, can significantly reduce multiple bone metastases of EMPD (45). Although the effect of CAB lasted only for 6 months, it did not cause severe bone marrow suppression; hence, androgen-deprivation therapy could be one of the potential therapeutic approaches for metastatic EMPD.

## Lymphangiogenesis and Epithelial–Mesenchymal Transition (EMT) of Paget Cells in EMPD

The clinical manifestation of erythematous, eczematous plaque of EMPD indicates the presence of vasodilation and hyperemia in the dermis of EMPD lesion. In fact, the histology of *in situ* and invasive EMPD lesions demonstrates a prominent enlargement and formation of several lymphatic and blood vessels in the dermis compared to those of healthy skin or other skin cancers such as melanoma (18). The immunohistochemical analysis established the expression of VEGF-A in Paget cells, as well as macrophages, and VEGF-C was also expressed in Paget cells (18). Both VEGF-A and VEGF-C are renowned cytokines related to angiogenesis and lymphangiogenesis, and evidence suggests that the generation of new blood and lymphatic vessels is crucial in cancer metastasis (46). Intriguingly, the immunohistochemical analysis also revealed that lymphatic endothelial cells (LECs) in a primary lesion express stromal cell-derived factor-1 (SDF-1), and N-cadherin-positive Paget cells co-expresses CXCR4, a specific ligand of SDF-1 (18). Notably, N-cadherin, vimentin, and snail are molecules that are upregulated in the EMT, a process through which epithelial cells lose their polarity, cell–cell adhesion, and E-cadherin expression, and TGF-β1 can induce EMT (47). A functional *in vitro* study using a human squamous carcinoma cell line corroborated with human pathological findings and revealed that snail increases the CXCR4 expression on tumor cells in the presence of TGF-β1 and the EMT process augments tumor cell migration through the CXCR4–SDF-1 axis (Figure 1B) (18). Furthermore, the CXCR4 expression of Paget cells in the primary lesion correlated with the presence of LN metastasis and reduced the disease-specific survival (18, 48). Likewise, the N-cadherin and vimentin expression of Paget cells in the primary lesion also correlated with the reduced OS (18). Hence, these findings implicate a crucial role of lymphangiogenesis and EMT of Paget cells in promoting LN metastasis of EMPD, and blocking the CXCR4–SDF-1 axis can be a novel option of adjuvant therapy for patients with CXCR4-positive invasive EMPD to prevent LN metastasis. Furthermore, the blockade of mutated PIK3CA or AKT1 might be an effective adjuvant therapy because the DNA sequencing of EMPD revealed a correlation of *PIK3CA* and *AKT1* mutations with E-cadherin hypermethylation (33).

## The Role of Receptor Activator of Nuclear Factor Kappa-B Ligand (RANKL)–Rank Interaction in the Tumor Microenvironment of EMPD

Although the aberrant activation of a signaling pathway in tumor cells and lymphangiogenesis mediates the progression of EMPD, the role of other cells, especially immune cells, in the tumor microenvironment of EMPD remains unclear. Histologically, EMPD exhibits abundant lymphocyte infiltration. The number of CD8^+^ T cell infiltration in noninvasive and invasive EMPD is similar; however, the number of CD8^+^ T cell-expressing granulysin and perforin is relatively lower in invasive EMPD than that of noninvasive EMPD, signifying the presence of stronger immunosuppression in the tumor microenvironment of invasive EMPD than noninvasive EMPD (49). Reportedly, invasive EMPD comprises a significantly higher number of CD163^+^Arg1^+^ M2 macrophages, an immunosuppressive macrophage, compared to noninvasive EMPD (49).

The RANKL and its receptor, RANK signaling, exerts numerous effects on immunity and promotes the survival of conventional dendritic cells and T-cell priming, thereby generating active immune responses (50). By contrast, it controls the number of regulatory T cells (Tregs) and induces tolerance against antigens (51). In EMPD lesions, Paget cells profoundly express RANKL with matrix metalloproteinase-7, which cleaves RANKL to release a soluble form (sRANKL), facilitating interaction with nearby cells expressing RANK (19). Remarkably, RANK is primarily expressed in CD163^+^Arg1^+^ M2 macrophages, and *in vitro* studies using monocyte-derived M2 macrophages have demonstrated that these cells produce CCL17 and promote the migration of CCR4-expressed CD4^+^ T cells, which comprise effector CD4^+^ T cells and Tregs when treated with sRANKL (20). In line with the *in vitro* experiment, CCL17 was co-expressed on the CD163^+^Arg1^+^ M2 macrophages in invasive EMPD (20). Furthermore, a study has reported that effector Tregs comprising a robust immunosuppressive effect profoundly express CCR4 (52) and that increased numbers of Tregs were related to more extensive cases of vulvar EMPD and disease recurrence (53). These studies highlight the crucial role of CD163^+^Arg1^+^ M2 macrophage and Tregs in establishing an immunosuppressive microenvironment in invasive EMPD by the RANKL–RANK interaction that promotes EMPD progression and causes poor prognosis (Figure 1B).

Reportedly, RANKL binds to RANK on osteoclasts and serves as a critical factor for regulating bone remodeling, and the activation of the RANKL–RANK signaling augments bone metastasis of various cancer types such as breast cancer (50). Based on this notion, a study demonstrated that denosumab, the anti-RANKL antibody, significantly delayed the appearance of skeletal-related events compared to bisphosphonates in patients with breast cancer with bone metastasis, thereby initiating its use to treat various metastatic bone tumors (54). Bone has been known as one of the most common site, where EMPD develops distant metastasis (55, 56). Based on these findings, denosumab seems not only useful for patients with metastatic EMPD and bone metastasis but also might be effective from the early stage to prevent the progression of invasive EMPD. Furthermore, since RANKL is related to immunosuppression of the EMPD microenvironment, denosumab could be a potential to enhance the efficacy of immunotherapy.

## Immunotherapy for Metastatic EMPD

Although the response rate is 20–35% in solid cancers, immunotherapy with anti-PD-1 antibody is a prominent therapeutic approach for cancers. This is because it can induce a durable response in a majority of responders for more than 2 years, which is uncommon with the molecular targeted therapy (57–60). The biomarkers that define anti-PD-1 antibody responders remain unclear; however, it was recently revealed that cancer with DNA mismatch-repair (MMR) gene mutations, so called “mismatch-repair-deficient cancer,” significantly responded better to anti-PD-1 antibody than MMR proficient cancer (61). In addition, it was reported that an anti PD-1 antibody induced robust antitumor immunity and attained durable disease control in heavily treated patients with colorectal cancer with MMR-deficient or microsatellite instability-high (MSI-H) status (62). Overall, these results propose that the MMR status or MSI-H is a useful biomarker to predict the clinical benefit of anti-PD-1 antibody, serving as the basis for the FDA’s approval of an anti-PD-1 antibody for unresectable or metastatic MMR-deficient or MSI-H cancers, irrespective of cancer’s original location.

The MMR pathway affects removal and correction of DNA base mismatches that arise either during DNA replication or caused by DNA damage (63). A mutation of genes involved in MMR, *MLH1, PMS1, MSH2*, and *MSH6* predispose to several tumorigenic conditions, such as Lynch syndrome, and cause cancer cells to display MSI-H (63). Although inactivation of MMR elevates the mutational burden, thereby promote carcinogenesis, a recent *in vivo* functional analysis revealed that it also leads to dynamic mutational profiles, resulting in the persistent renewal of mutation-associated neoantigens (MANAs) triggering durable immunosurveillance that can be further enhanced by an anti-PD-1 antibody. By contrast, MMR proficient cells exhibited stable mutational load and MANA profiles over time, which was consistent with the results of clinical trials (61, 64, 65).

Extramammary Paget’s disease has long been recognized to pose a high risk of developing secondary cancer. Reportedly, 14–32% of cases have also been diagnosed with other primary cancers (66, 67). Kang et al. hypothesized that *MMR* gene mutations could be associated with the high occurrence of secondary cancer in EMPD and investigated the MMR status and the presence of gene mutation in *MLH1, PMS1, MSH2*, and *MSH6* in 20 patients with EMPD (68). Their results revealed 8 of 20 cases (40%) with germline *MMR* genes missense mutations. Of these 8 cases with *MMR* genes mutations, 1 and 4 cases exhibited MSI-high or MSI-lo, respectively, whereas none of the other 12 cases without *MMR* genes mutations exhibited MSI. Furthermore, their sequencing analysis of *MLH1* and *MSH2* gene for 172 samples identified germline and somatic mutations of *MLH1* or *MSH2* in 34.3 and 13.4% of cases, respectively. Of these, *MLH1 V384D* (15.7%) and *MLH1 R217C* (4.1%) were top two germline mutations, and these detection rates were significantly higher than healthy controls. Furthermore, the functional *in vitro* assay revealed that *MLH1 V384D* and *MLH1 R217C* mutations had 50–60% MMR efficiency than wild-type *MLH1*. Although having high tumor mutational burden does not always associate with improved survival and clinical trials should be conducted to evaluate the survival benefit, these findings suggest that there is a decent percentage of MMR-deficient EMPD, which has an impaired MMR machinery, in EMPD and this subset of patients might achieve a durable response by anti-PD-1 immunotherapy.

## Conclusion

Metastatic EMPD is an aggressive skin adenocarcinoma with poor prognosis. Since current chemotherapeutic regimens are only moderately effective, improving clinical outcomes is imperative. The basic and translational research to date has provided an insight into the mechanisms promoting metastasis of EMPD that provide potential therapeutic targets for new drug development. Seemingly, Paget cells augment the ability of proliferation and survival by activating the RAS–RAF–MEK–ERK signaling, PI3K–AKT–mTOR signaling, or androgen–AR signaling. In addition, the interaction of Paget cells with other cells, such as LECs and CD163^+^Arg1^+^ macrophages in a tumor through the CXCR4–SDF-1 signaling and RANKL–RANK signaling, respectively, could establish a favorable tumor microenvironment to promote metastasis of Paget cells. Furthermore, recent genomic analysis of MMR has revealed that a decent percentage of EMPD comprises MMR-deficient EMPD cases that might achieve durable clinical response by an anti-PD-1 antibody. Hence, we are now beginning to understand multiple aspects involved in the pathogenesis of metastatic EMPD, and these findings will be sure to lead to better treatments for patients with metastatic EMPD in the future.

## Author Contributions

KF wrote the manuscript with input and guidance from TF.

## Conflict of Interest Statement

The authors declare that the research was conducted in the absence of any commercial or financial relationships that could be construed as a potential conflict of interest.
