# Corrigendum: Metastatic Extramammary Paget’s Disease: Pathogenesis and Novel Therapeutic Approach

**DOI:** 10.3389/fonc.2018.00047

**Published:** 2018-03-12

**Authors:** Keitaro Fukuda, Takeru Funakoshi

**Affiliations:** ^1^Department of Dermatology, University of Massachusetts Medical School, Worcester, MA, United States; ^2^Department of Dermatology, Keio University School of Medicine, Tokyo, Japan

**Keywords:** metastatic extramammary Paget’s disease, HER2–PI3K/ERK signaling, lymphangiogenesis, CXCR4–stromal cell-derived factor-1 axis, CD163^+^M2 macrophage, receptor activator of nuclear factor kappa-B ligand–RANK signaling, mismatch-repair deficient, anti-PD-1 antibody

In the published article, there was an error in the number of ongoing clinical trial for HER2-positive unresectable or metastatic EMPD “(UMIN-000006629)”. Instead of “(UMIN-000006629)”, it should be “(UMIN000021311)”. The authors apologize for this error and state that this does not change the scientific conclusions of the article in any way.

The original article has been updated.

## Conflict of Interest Statement

The authors declare that the research was conducted in the absence of any commercial or financial relationships that could be construed as a potential conflict of interest.

